# Pepper Alkaloid Piperine Increases Radiation Sensitivity of Cancer Cells from Glioblastoma and Hypopharynx In Vitro

**DOI:** 10.3390/ijms23158548

**Published:** 2022-08-01

**Authors:** Sascha Diehl, Guido Hildebrandt, Katrin Manda

**Affiliations:** Department of Radiotherapy and Radiation Oncology, University Medical Center Rostock, Suedring 75, 18059 Rostock, Germany; sascha.diehl@uni-rostock.de (S.D.); guido.hildebrandt@uni-rostock.de (G.H.)

**Keywords:** piperine, T98G, FaDu, ionizing irradiation, clonogenic survival

## Abstract

In our study, our aim was to examine the cytotoxic and radio-sensitizing effect of the alkaloid piperine, a major pungent of black pepper, on two different human epithelial tumor cell lines in vitro. The growth of the human cell lines T98G (glioblastoma) and FaDu (hypopharyngeal carcinoma) was examined under the influence of piperine in different concentrations. In addition, after combined treatment with ionizing radiation, long-term survival was investigated with a colony formation assay. The proliferation was analyzed using the BrdU-assay, while the DNA repair capacity was examined via the γH2AX assay. Piperine reduced the growth of both cell lines in a concentration-dependent manner as well as a time-dependent one. After combined treatment with piperine and ionizing radiation, an inhibition of clonogenic survival could be proven. A reduced proliferation capacity and an additive effect on DNA damage 24 h after irradiation are possible causal mechanisms, which were also demonstrated for both cell lines. Based on the results presented in this study, piperine was shown to have cytotoxic antitumor activity and a radio-sensitizing effect in micromolar concentrations in the human tumor cells that were tested. Based on these results piperine represents a potential therapeutic option in radio-oncological treatment.

## 1. Introduction

Owing to the increasing incidence of malignant tumor diseases, there is a great medical need for modern treatment options. An important strategy in today’s radiation oncology is the combination of radiation therapy with cytostatic or cytotoxic substances. In addition to the development of new therapeutic approaches, the search for active ingredients in nature still offers great potential. It was already possible in the past to obtain active ingredients from plants, such as taxanes, which are still used today in oncology, e.g., in combined radio-chemotherapy. Piperine is one such substance that has become the focus of interest in recent years because of its anti-tumor effects.

Piperine (1-piperoylpiperidine) is an alkaloid from plants of the *Piperaceae* family, which can be isolated from the fruits or roots of black pepper (*P. nigrum*) and long pepper (*P. longum*) [1]. Alkaloids are a group of organic compounds that, among other things, consist of a heterocyclic ring with a nitrogen atom and mostly have alkaline properties [2]. Piperine was first extracted in 1819 by Hans Christian Orsted. In addition to its use as a spice, the plant *Piper nigrum* has long played an important role in traditional phytomedicine and is mainly used for digestive and respiratory problems [3]. Its effect as a bioenhancer—which increases the bioavailability of active substances leading to increased bioefficacy—was described, whereby the effect of various medicinal substances can be increased. This is realized through an increase in gastrointestinal blood flow and an inhibition of P-glycoprotein, an ATP-dependent efflux pump in eukaryotic cells [4]. Recent investigations also point to an anti-tumorigenic effect of piperine. Various preclinical studies were able to demonstrate an inhibitory effect of piperine on the cell cycle. For example, in prostate and colon carcinoma cells, a cell cycle arrest at the transition of the G1/S phase on account of an increased expression of the CDK inhibitors p21^Cip1^ and p27^Kip1^ could be observed [5,6]. In addition, pro-apoptotic influences could be observed through piperine. In addition to the increased activation of effector caspase 3, a decrease in the anti-apoptotic protein Bcl-2 and an increased expression of the proteins Bax and caspase 9 could be detected in melanoma cells [7]. Furthermore, effects on the invasion or metastasis of malignant cells were shown by a reduced expression of matrix metalloproteinases in fibrosarcoma cells [8] and an inhibition of angiogenesis by suppressing the VEGF effect [9].

Hence, various in vitro analyses have already been able to show a large number of anti-tumorigenic starting points in different established cell lines for the active substance piperine, so that a combination with ionizing radiation appears promising. The aim of this work was therefore to investigate the combined effect of the alkaloid piperine and ionizing radiation on the human tumor cell lines T98G (glioblastoma) and FaDu (hypopharyngeal carcinoma). The cell lines examined are representative of in vivo tumor entities which, despite modern therapy options, are associated with a poor prognosis for the affected patients. An established treatment option for both tumor entities is radiation therapy. Additive or supra-additive effects of the active ingredient piperine could lead to a further reduction in the tumor burden and a reduction in radiation doses in order to minimize side effects for the patient.

## 2. Results

### 2.1. Inhibition of Cell Growth

**Figure 1:**
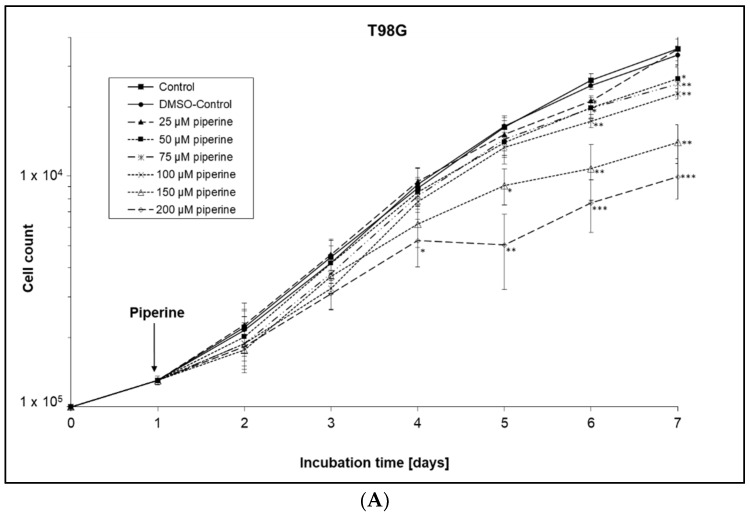

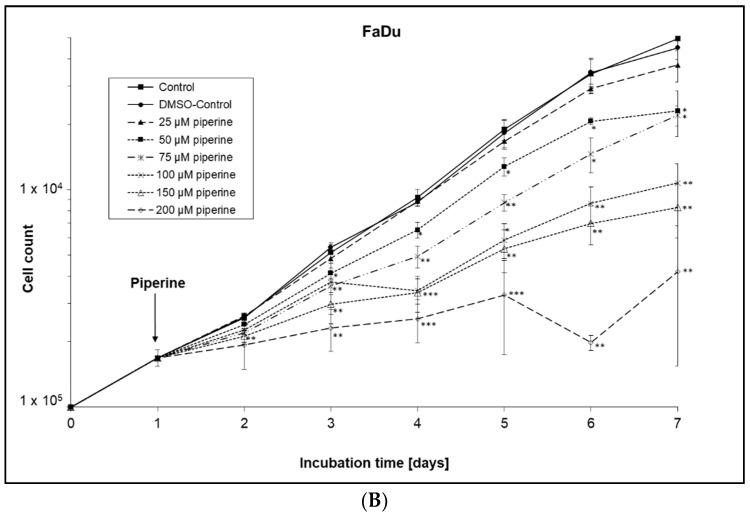
Growth curves of piperine-treated cells. The cells were seeded at day 0. Piperine was added to the cells at day 1 of incubation after a 24 h cell adhesion period to (**A**) T98G cells and (**B**) FaDu cells. Error bars represent the standard deviation of three separate experiments; wells were assayed in triplicates in each of the different experiments. Significance was calculated for each day’s approaches (control versus treated sample). Asterisks illustrate significances * *p* ≤ 0.05, ** *p* ≤ 0.01, *** *p* ≤ 0.001.

The growth curves without the influence of piperine showed an almost typical course. The population doubling time of T98G cells was 26.3 h. A population doubling time of 27.7 h in the exponential growth phase was determined for the FaDu cells. Dose- and time-dependent growth delays were demonstrated for both cell lines under the influence of piperine (Figure 1).

Three days after treatment with piperine, the T98G cells showed growth inhibition from a concentration of 150 µM, which was even statistically significant after the addition of 200 µM. Five days after the addition of piperine, an inhibitory effect on growth could also be demonstrated for lower piperine concentrations of 25 μM, 50 μM, 75 μM, and 100 μM. The IC_50_ value was 133 µM after a six-day incubation period. The growth-inhibiting effect was evident earlier in the FaDu cells than in the T98G cell line. The inhibition of growth was already clearly recognizable after a 48-h incubation from a piperine concentration of 50–75 µM and was statistically significant for the 75 µM concentration. After six days of incubation with piperine, the IC_50_ value was 74 µM. Thus, the FaDu cells were more growth sensitive to piperine than the T98G cells.

### 2.2. Clonogenic Survival

**Figure 2:**
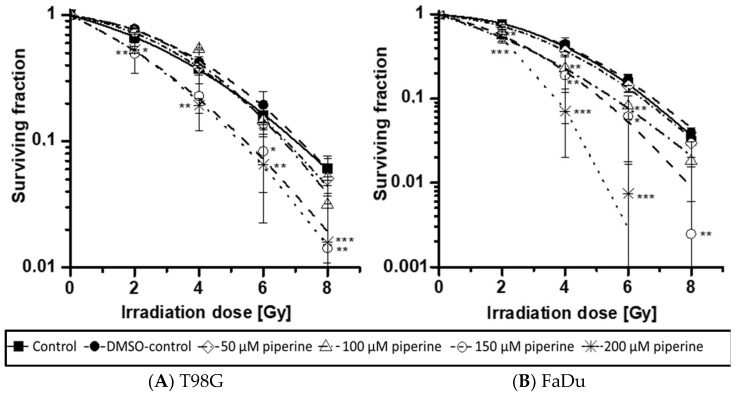
Clonogenic survival curves of piperine-treated (**A**) T98G cells and (**B**) FaDu cells in combination with ionizing irradiation (2 Gy–8 Gy) or non-irradiation (0 Gy). Piperine was added to the cells 24 h before irradiation; test duration was 12 days. Colonies were stained with crystal violet and counted manually by scoring only colonies with a minimum of 50 cells. The surviving fractions of treated cells were normalized to the plating efficiency of untreated controls (0 µM piperine, 0 Gy). Error bars represent the standard deviation of at least three separate experiments (T98G: control: *n* = 7; DMSO-control, 50 µM piperine, 100 µM piperine: *n* = 3; 150 µM piperine, 200 µM piperine: *n* = 4; FaDu: control: *n* = 7; DMSO-control, 50 µM piperine, 100 µM piperine: *n* = 4; 150 µM piperine, 200 µM piperine: *n* = 3). Asterisks illustrate significances * *p* ≤ 0.05, ** *p* ≤ 0.01, *** *p* ≤ 0.001 related to the control of the respective radiation dose.

In the cells treated without the drug, an increasing radiation dose resulted in a decrease in survival in both cell lines (Figure 2). The proportion of clonogenic cells after irradiation with 2 Gy can be given as an SF_2_ value and serves as a measure of the radiation sensitivity of a cell line. An SF_2_ value of 0.66 was determined for the T98G cells; 0.75 was calculated for the FaDu cells. The solvent DMSO had no effect on the clonogenic survival of either cell line. The influence of the active ingredient piperine was already evident in the morphology of the cell colonies.

**Figure 3:**
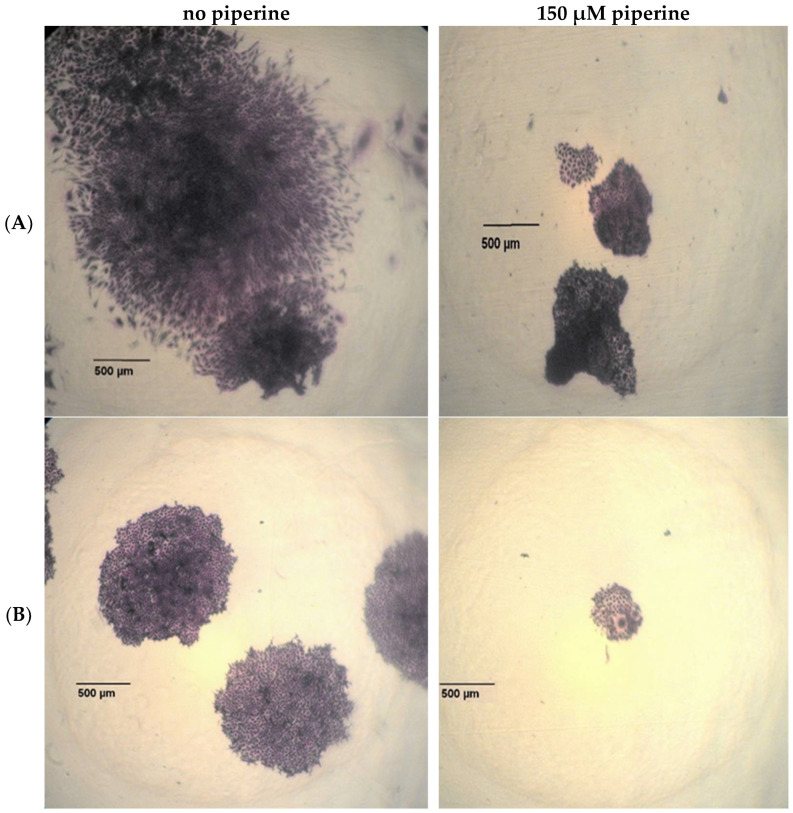
Microscopic representation of the colony morphology of unirradiated (**A**) T98G cells and (**B**) FaDu cells with and without the addition of piperine with a concentration of 150 μM. 40× magnification.

Microscopically, the T98G cells lost their fibroblast-like shape and formed significantly more compact colonies (Figure 3A). The cell colonies of the FaDu cells also showed smaller and rather more compact colonies after the addition of the piperine (Figure 3B). After adding the drug piperine, synergistic effects from the combination of radiation and piperine could be observed on the basis of the cell survival curves (Figure 2A,B). For both cell lines, a radiation-sensitizing effect could be demonstrated by piperine in higher concentrations. The hypopharyngeal carcinoma cell line FaDu already showed significant radio sensitization after irradiation with 2 Gy and under the influence of 150 µM and 200 µM piperine (Figure 2B). If the radiation dose was increased to 4 Gy, a radiation-sensitizing effect was statistically significant from an active substance concentration of 100 µM. For the T98G cell line, a significant reduction in cell survival was determined after the addition of 150 µM and 200 µM and after irradiation with 2 Gy (Figure 2A). Despite an increase in the radiation dose, lower piperine concentrations had no statistically significant effect on the clonogenic survival of the T98G cells.

### 2.3. Cell Proliferation

**Figure 4:**
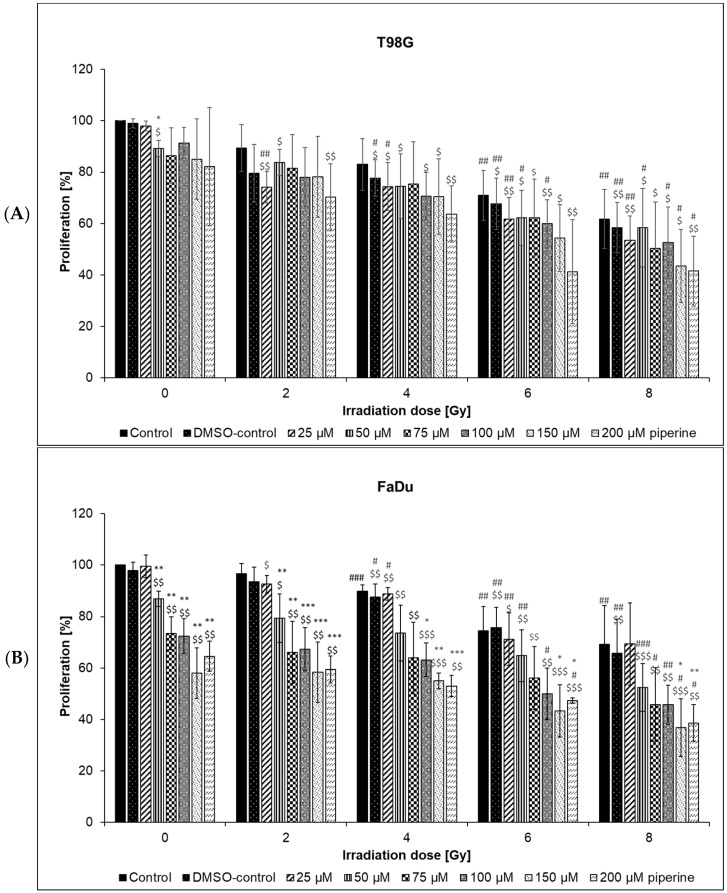
Proliferation of (**A**) T98G cells and (**B**) FaDu cells treated with piperine in combination with ionizing radiation (2–8 Gy) and non-irradiated controls (0 Gy) measured by BrdU-assay. Piperine was added to the cells 24 h after seeding and 24 h before irradiation. The proliferation of treated cells was normalized to the values of untreated controls (0 µM piperine, 0 Gy; 100%). Error bars represent the standard deviation of at least three separate experiments; wells were assayed in six replicates in each of the different experiments. Significances were calculated for experiments (^$^) related to non-irradiated control (0 µM piperine; 0 Gy), (*) related to control (0 µM piperine) of the same radiation dose, (#) related to the unirradiated sample with the same piperine concentration. Asterisks/hash signs/dollar signs illustrate significances *^/#/$^ *p* ≤ 0.05, **^/##/$$^ *p* ≤ 0.01, ***^/###/$$$^ *p* ≤ 0.001.

Proliferation was assessed using the BrdU assay. Proliferation-inhibiting effects were found in both cell lines examined after irradiation alone (Figure 4).

ANOVA analysis revealed significant dose- and concentration-dependent significant changes for all proliferations at all radiation doses, compared with untreated corresponding controls. In the T98G cell line, the proliferation of the untreated control (100%) was statistically significantly reduced to 71% after a radiation dose of 6 Gy (Figure 4A). In the FaDu cells, there was a significant inhibition of proliferation to 89% from a dose of 4 Gy (Figure 4B). After irradiation with 6 Gy, a proliferation of 75% could be calculated in the FaDu cells compared with the non-irradiated control. A proliferation-inhibiting effect of piperine could be demonstrated for both cell lines, with this being more pronounced in the FaDu cell line. After addition of the active substance piperine in a concentration of 50 µM, the proliferation in both investigated cell lines was significantly reduced. A further significant reduction in proliferation could not be detected in the T98G cell line because of the high standard deviation. In contrast, a clear dose–response relationship was found in the FaDu cells. For example, after the addition of 150 µM piperine, proliferation was significantly reduced to 58% compared with the untreated control (100%). The combined treatment with ionizing radiation and the active ingredient piperine had a synergistic effect on both cell lines with regard to proliferation inhibition. For example, in the FaDu cell line, after irradiation with 2 Gy under the influence of 200 µM piperine, proliferation was reduced by 42%. However, if the FaDu cells were irradiated only with a dose of 2 Gy, the inhibition of proliferation was only 3%, and after treatment with the 200 µM active substance alone, it was 35%. By increasing the radiation dose to 8 Gy and under the influence of the same drug concentration, proliferation was even reduced by 61% compared with the untreated control. This synergistic effect was also evident in the T98G cell line, but from a piperine concentration of 150 µM and an irradiation dose of 6 Gy, proliferation could not be reduced further by increasing the concentration of the active substance or the irradiation dose.

### 2.4. Detection of DNA Double-Strand Breaks

**Figure 5:**
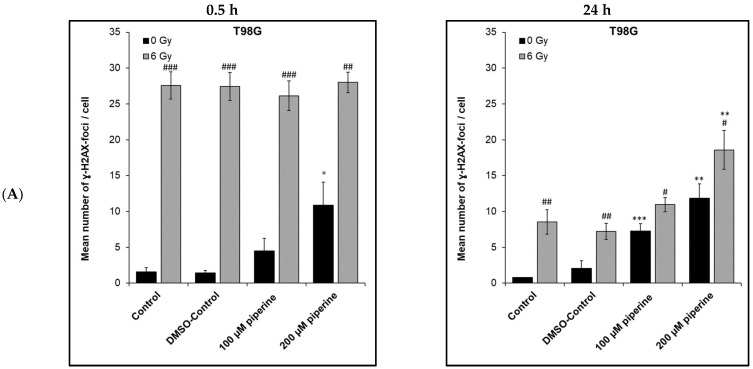

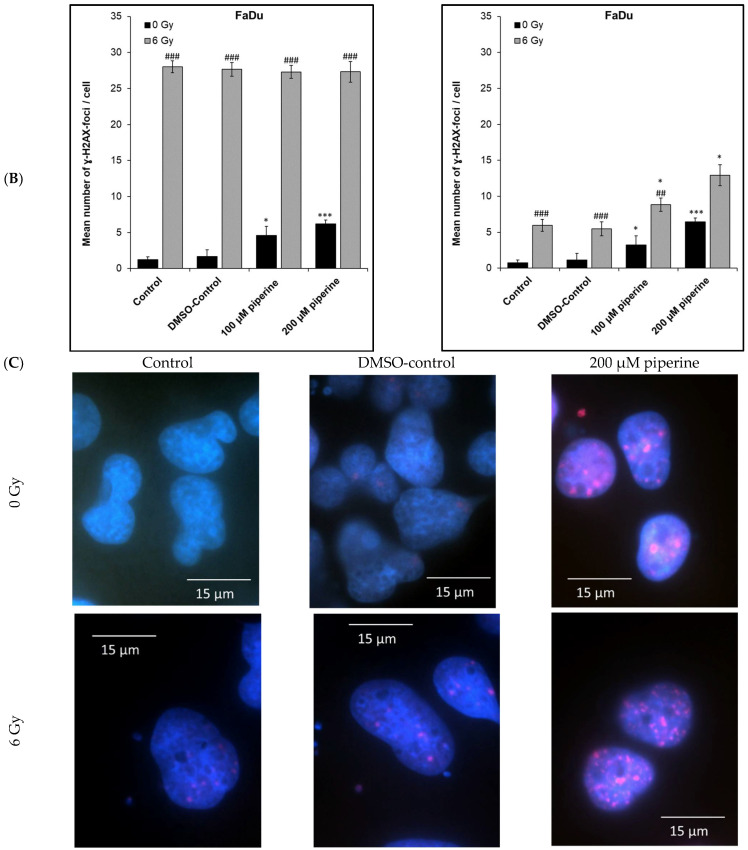
Effect of piperine on the number of DNA double-stand breaks (DSB) and their repair capacity in (**A**) T98G cells and (**B**) FaDu cells after repair times of 0.5 h or 24 h, respectively, detected by γ-H2AX-assay. Error bars indicate the standard deviation for three independent experiments. Significances were calculated in relation to the control of the respective radiation dose (for non-irradiated experiments: 0 µM piperine; 0 Gy; for irradiated experiments: 0 µM piperine; 6 Gy) and illustrated by asterisks (* *p* ≤ 0.05, ** *p* ≤ 0.01, *** *p* ≤ 0.001). Hash signs illustrate significances between data from the non-irradiated approach of the same piperine concentration (^#^ *p* ≤ 0.05, ^##^ *p* ≤ 0.01, ^###^ *p* ≤ 0.001). (**C**) Depiction of the T98G cell nuclei using DAPI (blue) and γ-H2AX (red) staining; 1000× magnification.

Using the γ-H2AX test, it was possible to analyze the number of DNA double-strand breaks and, hence, their repair capacity in T98G and FaDu cells after ionizing radiation or/and under the influence of piperine. In both cell lines, a small number of DNA double-strand breaks was already noticeable in the control batches, which were neither irradiated nor treated with the active substance. On average, 1–2 foci per cell nucleus could be counted in the control samples (Figure 5). 

After irradiation with 6 Gy alone, there was a significant increase in the number of DNA double-strand breaks in both tumor cell lines compared with the non-irradiated control. Already, 0.5 h after radiation exposure, an average of 28 DSB per cell nucleus could be determined in both the T98G and FaDu cells, with a maximum of 30 DSB/cell nucleus being counted. Significant differences between the individual batches within the same cell line could, therefore, not be observed. Piperine without ionizing radiation led to a concentration-dependent increase in the number of DNA double-strand breaks. The T98G cells under the influence of the 200 µM active substance were more sensitive than the FaDu cells (approx. 6 DSB/cell nucleus) at both fixation times with an average of approx. 11–12 DSB. Even with 100 µM piperine, a significant increase in DNA double-strand breaks could be seen in both cell lines. Under the combination of radiation and piperine, a maximum of 30 DNA-DSB/cell nucleus were counted. The DNA damage after irradiation with 6 Gy was so pronounced that, with this maximum number of foci counted, no additional foci as differences between the batches could be detected even after the addition of piperine. 

After 24 h, the number of residual foci of piperine-induced DNA-DSB was unchanged compared with 0.5 h. Thus, no repair of this genotoxic damage caused by piperine took place. However, notes for an additive effect after combined treatment with irradiation and piperine could be observed. At this time point, the repair of the DNA-DSB is already complete. The number of residual foci of the irradiated cells was significantly higher under the influence of piperine than without the active substance. Significant results could be observed for both cell lines after treatment with 100 µm and 200 µM piperine. Without piperine, in irradiated cells nine DSB/cell could be counted in the T98G cell line. In contrast, after the addition of 200 μM piperine, in irradiated cells 19 DSB/cell were detected. Similar results were obtained for irradiated cells of the FaDu cell line: 6 DSB/cell were counted without the influence of the active substance, whereas 13 DSB/cell were visible after treatment with 200 µM piperine. In both cell lines, the repair capacity of radiation-induced DNA-DSB was reduced under the influence of piperine; additive effects were observed.

## 3. Discussion

The aim of this study was to investigate the combined effect of the alkaloid piperine and ionizing radiation on the human tumor cell lines T98G (glioblastoma) and FaDu (hypopharyngeal carcinoma). Based on the available data, dose- and time-dependent growth retardations under the influence of piperine were demonstrated for both cell lines, although the cell line FaDu was slightly more sensitive. Piperine-mediated inhibition of cell growth was already described for other human cell types or, for example, for different breast cancer cells [10], prostate cancer cells [5], bronchial cancer [11], and for another oropharyngeal carcinoma cell line KB [12]. Various working groups came to the conclusion that the growth-inhibiting and cytotoxic effect of piperine has several causes. An apoptosis-inducing effect of piperine has often been described in the literature, with piperine appearing to influence both the extrinsic and the intrinsic signaling pathway of apoptosis. Among other things, increased activities of caspase 3 and 9, e.g., in ovarian carcinoma cells [13] and in colon carcinoma cells HT-29 [14], could be detected after the addition of piperine. Furthermore, the intrinsic mitochondrial signaling pathway of programmed cell death was shown to be altered in mammary carcinoma cell lines. An increase in pro-apoptotic proteins such as Bax (Bcl-2 associated X protein) could be detected [15]. Subsequently, there was also an increase in other pro-apoptotic molecules such as cytochrome c and Smac/DIABLO, which have caspase-activating effects [10]. For the aforementioned oropharyngeal carcinoma cell line KB, an increased formation of reactive oxygen species could be detected, which led to a loss of mitochondrial potential and consequently programmed cell death [12]. In addition to this apoptosis induction, cell cycle arrest was also observed, both at the restriction point of the G2/M phase in MCF-7 cells [15] and at the transition from the G0 to the G1 phase in prostate carcinoma cells [16], which caused cellular senescence or initiation of apoptosis.

To assess the influence of piperine on the cellular radiation response, the clonogenic survival of cells was determined using clinically relevant doses from 2 Gy to 8 Gy. The cell survival curves obtained from the results of the colony-forming test allow conclusions to be drawn about the radiation sensitivity of the cells. In the cells treated without piperine, increasing the radiation dose resulted in a decrease in survival in both cell lines. A dose–response relationship with regard to treatment with ionizing radiation could, therefore, be demonstrated for both the T98G and the FaDu cells. After adding the drug piperine, synergistic effects from the combination of radiation and piperine could be observed on the basis of the cell survival curves. For both cell lines, a radiation-sensitizing effect could be demonstrated by piperine in higher concentrations. The FaDu cell line showed these effects from a lower piperine concentration than the T98G cell line and was, therefore, again more sensitive. Ionizing radiation or other toxic agents can lead to the loss of the reproductive integrity of the tumor cells and thereby cause clonogenic cell death, which is multifactorial. Investigations regarding a radio-sensitizing effect by piperine have not yet been published for the two investigated cell lines T98G and FaDu. However, an increased radiation effect was observed for the breast carcinoma cell line MDA-MB-468 [10]. In these investigations, a radiation-sensitizing effect after doses of 4 Gy and 6 Gy was found even after treatment with 25 µM piperine. Similar experiments were also carried out for the HT-29 cell line of a colorectal carcinoma [17]. This established cell line also showed increased radiosensitivity after piperine treatment. A cell cycle arrest at the transition from the G2 to the M phase could be proven as the causative mechanism. This so-called G2/M block represents a known biological effect of ionizing radiation [18]. The duration of this temporary block depends on the cell line and the radiation dose. If the cells are not able to repair the radiation-induced damage, programmed cell death can be initiated. Increased apoptosis, especially of the mitochondrially mediated pathway, has been discussed as another radio-sensitizing mechanism by piperine. An upregulation of pro-apoptotic proteins such as Bax and a reduced expression of the anti-apoptotic Bcl-2 protein could be demonstrated [17]. Studies on the murine melanoma cell line B16F10, in which apoptosis was caused by an increased formation of reactive oxygen species, provided comparable results [19]. The formation of oxygen radicals after exposure to ionizing radiation represents an important radiobiological effect. Reactive oxygen species damage all kinds of molecules. Strand breaks within the DNA molecule, protein modifications, and oxidation of lipid molecules, these processes are summarized as oxidative stress. Even after treatment with piperine, an increased intracellular concentration of reactive oxygen species could be detected [19], so that additive effects on the clonogenic survival of the cells are possible in combination with ionizing radiation.

By examining cell proliferation using the BrdU assay, the causal mechanisms—for example, of the results observed using growth curves and colony formation tests—could be further classified. Irradiation alone, as well as piperine from a concentration of 50 µM, resulted in an inhibition of proliferation. A combination effect of piperine and ionizing radiation was detectable for both cell lines. Proliferation-inhibiting effects through ionizing radiation have already been described for both the cell lines that were examined, whereby a dose dependency was also shown here; both the FaDu and the T98G cells can be classified as relatively radiation-resistant cell lines [20,21]. A proliferation-inhibiting effect of piperine could also be demonstrated for both cell lines, with this being more pronounced in the FaDu cell line. These results support the observations from the growth experiments. Various working groups have already been able to confirm that piperine inhibits the proliferation capacity in various cell lines. This resulted in both G1 and G2/M arrests. In human melanoma cells (SK MEL 28), a blockade of the cell cycle in the G1 phase was found under the influence of piperine. Reduced expression of cyclin D and E2F-1 and increased detection of the CDK inhibitor p21^Cip1^ were described as possible causal mechanisms [22]. Similar results were obtained from studies on the HT-29 cell line [6]. These investigations also showed a prolonged blockade of the cell cycle after the G1 phase, which was associated with a reduced expression of the cyclin-dependent kinases CDK4 and CDK6 and an increased expression of the CDK inhibitors p21^Cip1^ and p27^Kip1^. Hence, the G1 arrest induced by piperine represents a possible cause for the inhibition of proliferation measured in this work. The combined treatment with ionizing radiation and the active ingredient piperine had a synergistic effect on both cell lines with regard to proliferation inhibition. This also corresponds to the observations of two other working groups. In the human colon carcinoma cell line HT-29, for example, after the addition of 25 µg/mL piperine and after irradiation with only 1.25 Gy, the relative proliferation was reduced by almost 60% compared with the untreated control [17]. Synergistic effects were also found in the murine colon carcinoma cell line CT26, in which piperine increased the radiation effect by 20% [21].

Induced DNA double-strand breaks represent the most important radiobiological effect of ionizing radiation. The influence of piperine on the number of DNA-DSB and their repair capacity was investigated using the γ-H2AX assay. Under the sole influence of piperine, an increased number of DNA double-strand breaks could be determined both in the T98G and in the FaDu cells. The genotoxic effects of the alkaloid piperine appear to be manifold. Investigations of the human melanoma cell line SK MEL 28 revealed dose- and time-dependent increased levels of reactive oxygen species after the addition of piperine. In particular, in the S phase, these led to strand breaks during DNA replication and hence to increased recruitment of γ-H2AX [22]. Furthermore, a cell cycle arrest—both in the G1 and G2 phases—could be observed by piperine in various cell lines. In this context, Fofaria et al., (2014) demonstrated a G1 arrest through reduced expression of cyclin D1 and induction of the cell cycle inhibitor p21^Cip1^. This cell cycle arrest was associated with increased DNA damage and increased apoptosis. Twenty-four hours after irradiation, the repair of the DNA-DSB is already complete and the number of residual foci can be detected to make a statement for the repair capacity of the cells. Notes for an additive effect after combined treatment with irradiation and piperine could be observed at this time point. Under the influence of the two drug concentrations examined, significantly more residual DNA double-strand breaks were found 24 h after irradiation compared with the irradiated control. This effect increased with increasing drug concentration. 

Both examined cell lines in our study are characterized by a mutation in the p53 tumor suppressor (FaDu: p.R248L; T98G: p.M237I) [23]. The two cell lines, SK-MEL-28 as well as HT-29, in which piperine was also shown to have an effect [17,22], are also p53-mutated (SK-MEL-28: p.L145R; HT-29: p.R273H) [24]. However, it cannot be generally concluded from this that the combined effect of piperine and radiation is only present in p53-mutated cells or that there is no effect in wild-type cells. This is also countered by the fact that Tak et al., (2012) observed the effect of piperine in combination with irradiation on the colon carcinoma CT26 line [19], which corresponds to the wild type [25].

In conclusion, this study demonstrates the antiproliferative and radio-sensitizing effects of piperine. The drug causes a significant increase in the radiation response in the cells. This finding gives crucial evidence of the high potential of piperine for use in combined radio-chemotherapy in the future. Next investigations are following to get deeper insights into the mechanism of the radio-sensitizing effect of piperine.

## 4. Materials and Methods

### 4.1. Materials

Dulbecco’s modified Eagle’s medium (DMEM) was purchased from Lonza Group Ltd. (Walkersville, MD, USA). Phosphate buffered saline (PBS) was obtained from PAN BioTech (Aidenbach, Germany) and fetal bovine serum (FBS) from Biochrom AG (Berlin, Germany). Penicillin/streptomycin (100 U/mL/100 µg/mL) and piperine (>97%) were purchased from Sigma-Aldrich GmbH (Taufkirchen, Germany). 4-(2-hydroxyethyl)-1-piperazineethanesulfonic acid (HEPES), non-essential amino acids (NEA), and sodium pyruvate were purchased from ThermoFisher Scientific GmbH (Dreieich, Germany). Trypsin/EDTA (0.05%/0.25%) was obtained from PAA Laboratories GmbH (Cölbe, Germany). Piperine was dissolved in dimethylsulfoxide (DMSO; Carl Roth GmbH and Co. KG, Karlsruhe, Germany) to obtain a 150 mM stock solution. For experiments, piperine stock solution was diluted with DMEM to produce the final concentrations.

### 4.2. Cell Culture

T98G and FaDu cells were purchased from American Type Culture Collection (ATCC, Manassas, VA, USA). The cell lines were cultivated at 37 °C, 5% CO_2_ in DMEM, supplemented with 10% FBS, and 1% penicillin/streptomycin. In addition, 1% NEA and sodium pyruvate and 2% HEPES were added to the medium of the FaDu cells. Both cell lines were passaged twice weekly to ensure exponential growth.

### 4.3. Irradiation

The cells were irradiated in cell culture flasks or in multi-well plates at room temperature using a Versa HD linear accelerator from Elekta Ltd. (Crawley, Great Britain). The irradiation was carried out with a nominal photon energy of 6 MV and a dose rate of 6.6 ± 0.6 Gy/min with doses of 2 Gy, 4 Gy, 6 Gy, or 8 Gy. A control sample (0 Gy) was transported to the radiation room to ensure comparable environmental factors.

### 4.4. Growth Curves

The cells were seeded in a 24-well plate in triplicates, with a seeding density of 1 × 10^4^ cells per well, for each experimental approach. Twenty-four hours after seeding, piperine was added in concentrations of 25 μM, 50 μM, 75 μM, 100 μM, 150 μM, or 200 μM respectively. A 1:100 dilution of the piperine stock solution was prepared for the concentrations of 25–75 µM, and a 1:10 dilution for the concentrations of 100–200 µM. In addition, the solvent was checked by adding DMSO (0.1%). On the day the piperine was added, the cell count of the cells seeded on the previous day was determined. In the following six days, the cell counts for all drug concentrations as well as the DMSO control and the untreated (native) growth control were then determined in triplicates. Growth curves were created on the mean of three independent experiments.

### 4.5. Clonogenic Assay

The colony formation test was carried out with a cell density of 2 × 10^3^ cells/T25 cell culture flask. The active substance was added 24 h after cell seeding (50 μM, 100 μM, 150 μM, and 200 μM). In addition, a control without active substance and a solvent control with DMSO were carried out. After a further 24 h, the cells were irradiated using a linear accelerator with doses of 0 Gy (control), 2 Gy, 4 Gy, 6 Gy, and 8 Gy. Seven days after the start of the experiment, the medium and the active substance dissolved in it were removed and a new active substance-free complete medium was added. After a further five days of incubation, the cells were fixed and stained. For this purpose, the medium was discarded and the cells were then fixed with 5 mL ethanol (70%). The cell colonies were stained with 1% crystal violet solution. First, the size and cell number of the colonies were assessed under the phase contrast microscope. Only colonies of at least 50 cells in size were counted. The counting then took place with the help of the light plate.

### 4.6. Proliferation

Proliferation was assessed using the BrdU assay. A 96-well plate was used to carry out the experiment. A quadruple determination was carried out for each batch. The 2 × 10^3^ cells were seeded into each well. After 24 h, the piperine was added in different concentrations (0 μM, 25 μM, 50 μM, 75 μM, 100 μM, 150 μM, and 200 μM). After an incubation of a further 24 h, BrdU was added (20 μL/well). Two hours later, the cells were irradiated using a linear accelerator (0 Gy, 2 Gy, 4 Gy, 6 Gy, 8 Gy). BrdU incorporation was measured another 24 h after irradiation. The further procedure was based on the information provided by the cell proliferation kit (Roche Diagnostics GmbH, Mannheim, Germany). The absorption was then measured at a wavelength of 450 nm on the spectral photometer.

### 4.7. Detection of DNA Double-Strand Breaks (γH2AX Assay)

Chambered coverslips with four chambers (LabTekfi, Nunc, Roskilde, Denmark) were used for the assay. The test was carried out in duplicate for each experiment. Twenty-four hours after cell seeding, piperine was added (100 μM or 200 μM). After a further 24 h incubation time, irradiation was carried out using a linear accelerator with 6 Gy. The cells were fixed 30 min or 24 h after irradiation. The maximum of DNA double-strand breaks can be seen about 30–90 min after the damage event, which is why the first fixation point in this work was chosen after 0.5 h. The 24 h point in time was also examined, since the repair of radiation-induced DNA-DSB is then completed and conclusions can be drawn about the repair capacity. The detached slides were then fixed and stained in accordance with the protocol already described [24]. The foci of a total of 50 cell nuclei were counted for each experiment using the fluorescence microscope. The mean value was then formed from the counts of the duplicates and evaluated as the result of a single experiment. A total of three independent experiments was carried out.

### 4.8. Statistical Analysis

Calculations were performed on the mean of at least three independent experiments. After examination on normal distribution, statistical analyses were carried out using Student’s *t*-test. *p* ≤ 0.05 was considered as the statistically significant difference. To compare a variable under different conditions (γH2AX assay), ANOVA analysis followed by a Bonferroni post test with SigmaPlot (Version 13.0, Systat Software GmbH) was utilized. There was no adjustment for multiple testing, since all experiments were carried out individually, and were, therefore, considered independently of one another.

## Data Availability

The data presented in this study are available on request from the corresponding author.

## Abbreviations

BrdU	Bromodeoxyuridine
DMEM	Dulbecco’s Modified Eagle´s Medium
DMSO	dimethylsulfoxide
DSB	double strand breaks
FBS	fetal bovine serum
PBS	phosphate buffered saline
